# 

**Published:** 1869-10

**Authors:** 


### CONTENTS:

TRANSLATIONS:
Investigations into the Pacinian Corpuscles, - - - - - - 557
Investigations into the Terminations of the Cutaneous Nerves in Man, - 562
HOSPITAL REPORTS:
Cook County Hospital, - - - 565
St. Luke's Hospital, - - - 575
EDITOR'S BOOK TABLE, - - - 578
FOREIGN ITEMS, - - - - - 586
PUBLISHERS' NOTICE, - - - 594
EDITORIAL:
Change of Publisher, - - - 590
Water Melon in Dysentery, - 590
Rush Medical College, - - - 594
EDITORIAL NOTES:
American Pharmaceutical Associat'n, 595
LOOT:
Treatment in Diarrhœa of Infants, 597
Diseased Gums, - - - - - 598
Rich Beef Tea, - - - - - 599
Treatment of Dysentery, - - 599
Carbolic Acid, - - - - 600
Ointment for Hæmorrhoids, - 600
Cure for Warts, - - - - 601

LOOT—Continued:
Sulphate of Soda in Chronic Cystitis, 601
Goitre, - - - - - - 601
Calabar Bean in Epilepsy, - - 601
" " Tetanus, &c., 605
Morphia in Sunstroke, - - - 602
Spasmodic Asthma of Children, - 603
Chronic Catarrh, - - - - 603
Muriate of Ammonia as a Remedy, 603
Lime Water in Bright's Disease, - 604
Strychnia in Amblyopia, - - 605
Preservation of Dead Bodies, – 605
A New Styptic Collodion, - - 605
Sulphophenic Acid, . - - 606
Starchy Food for Infants, - - 608
Antiseptic Treatment of Wounds, 610
Colotomy in Cancer of Rectum, with Stricture, - - - - - - 614
Chloral, - - - - - - - 618
Sleeplessness of Infants, - - 619
Parasitic Diseases of the Head, - 619
Animal Vaccination & Vaccinal Syphilis, 620
Medical Journal Advertiser.
W. B. Keen and Cooke's Catalogue of Medical Books.

